# Disease activity at the onset of diagnosis as a predictor of disease outcomes in a cohort of patients with systemic lupus erythematosus: A post hoc retrospective analysis of the COMOSLE-EGYPT study

**DOI:** 10.1007/s10067-024-07222-w

**Published:** 2024-11-16

**Authors:** Abir Mokbel, Nermeen A. Fouad, Alkhateeb Alkemary, Marwa Abdo

**Affiliations:** 1 Cairo University, Cairo, Egypt; 2 Fayoum University, Fayoum, Egypt

**Keywords:** Comorbidities, Damage, Mortality, SLEDAI

## Abstract

**Introduction:**

Systemic lupus erythematosus (SLE) has a non-uniform course directly reflected in changes in disease activity and anticipation of damage.

**Aim:**

To determine the impact of disease activity at the onset of disease diagnosis, measured by the Systemic Lupus Erythematosus Disease Activity Index (SLEDAI) disease activity score, on different disease parameters and outcomes.

**Methods:**

This multicentre, retrospective cohort study included 823 SLE patients. Disease damage was measured by the Systemic Lupus International Collaborating Clinics Damage Index (SLICC), and comorbidities were measured by the Charlson Comorbidity Index (CCI). According to the mean SLEDAI at onset of disease diagnosis, patients were classified into two groups: I included patients with a mean SLEDAI ≤ 10 (non-severe disease activity), and II included patients with a mean SLEDAI > 10 (severe disease activity).

**Results:**

SLEDAI at onset of disease diagnosis was a predictor of damage and comorbidities.

**Conclusion:**

A higher SLEDAI score at onset of disease diagnosis was associated with damage accrual. Patients who are younger at disease onset are more likely to have more severe disease. Severe disease activity at the onset of disease diagnosis was also associated with future comorbidity occurrences, but it was not significantly associated with mortality. SLEDAI at the onset of disease diagnosis could be a prognostic marker predicting the damage, which may help in the identification of patients who are at higher risk of adverse outcomes. Special care should be directed towards patients who are younger at disease onset as they may have a higher disease activity at diagnosis.

**Table:** 

**Key Points** • *This is a unique study as it is the first to focus on the impact of SLE disease activity at the onset of disease diagnosis measured by SLEDAI disease activity score on different disease parameters and outcomes.* • *Previous studies, though these are scarce, have highlighted the impact of disease activity throughout the disease course and not specifically at the beginning of the SLE disease.*

## Introduction

Systemic lupus erythematosus (SLE) is an autoimmune systemic disease with a relapsing–remitting course. Its presentation is heterogeneous; so many challenges exist for its diagnosis and management [1, 2]. Despite earlier diagnosis and treatment advances for SLE, the increased mortality and morbidity experienced by patients with SLE are still a major concern, indicating that several unmet needs are present among this group of patients, such as persistent disease activity and flares, dependence on glucocorticoid treatment, the burden of comorbidities, reduced quality of life, access to high-quality health care and damage accrual [3–5].

It is clinically important to determine the baseline characteristics that predict future severe disease and to identify modifiable factors that might affect disease outcomes [6]. Early identification of patients designated for more aggressive disease courses enables more timely therapeutic intervention and better disease control. Long disease quiescence was predicted by baseline low disease activity, the use of hydroxychloroquine and higher education. On the other hand, African American ethnicity and high disease activity in the early disease course could predict chronic activity in patients with SLE [7].

The non-uniform course of SLE is directly reflected in the changing state of disease activity over time [8]. Lupus activity can be defined as the sum of all clinical manifestations and serological abnormalities related to the ongoing immune-inflammatory pathways involved in SLE [9]. SLE disease activity could be measured by using the Systemic Lupus Erythematosus Disease Activity Index (SLEDAI) score. Several studies have shown that chronic disease activity, particularly during the early disease course, could anticipate the occurrence of damage in patients with SLE [10, 11]. In this study, we aimed to determine the impact of disease activity at the onset of disease diagnosison different disease parameters and outcomes. Previous studies, which are scarce, have highlighted the impact of disease activity through the disease course, but we focused on the disease activity at the disease onset.

## Materials and methods

This study is a post hoc analysis of the COMOSLE-EGYPT study, a multicentre, retrospective cohort study that included patients attending rheumatology units in four university hospitals in Egypt (Cairo, Beni-Suef, Menia and Fayoum) in addition to a private centre in Fayoum [12]. The study was conducted following the guidelines of the Declaration of Helsinki and was approved by the Ethics Committee of Biomedical Research of the Faculty of Medicine, Fayoum University (N: R494). The recruited patients fulfilled the 1997 American College of Rheumatology (ACR) classification criteria for SLE [13].

## Data collection

Data on patients included in this study were extracted from the COMOSLE-EGYPT study database after the approval of the study members. Demographic data, clinical manifestations and disease activity at the onset of disease diagnosis were measured by SLEDAI, which is a universal index composed of 24 weighted clinical and laboratory variables that reflect disease activity in the previous 10 days [14], disease damage measured by the Systemic Lupus International Collaborating Clinics damage index (SLICC) [15], comorbidities measured by the Charlson Comorbidity Index (CCI) [16], medications used and mortality were extracted. Patients with incomplete data for this ancillary study were excluded.

According to the mean SLEDAI at the onset of disease diagnosis, patients were classified into two groups: group I consisted of patients with a mean SLEDAI score ≤ 10 (non-severe disease activity), and group II consisted of patients with a mean SLEDAI score > 10 (severe disease activity) [14].

## Statistical analysis

The data were collected, tabulated and statistically analysed using SPSS software (IBM Corp., 2011). IBM SPSS Statistics for Windows, Version 20.0. (Armonk, NY, USA: IBM Corp.). Quantitative data are expressed as the *mean* ± *standard deviation* when normally distributed or as the median and range when not normally distributed. Qualitative data are expressed as numbers (percentages). Student’s *t*-test was used to analyse the difference between two independent groups for parametric data, and the Mann–Whitney *U*-test was used when the data were non-parametric.

The percentages of categorical variables were compared using the chi-square test or Fisher’s exact test when appropriate. Correlation analysis was used to find the relationship between SLEDAI at the onset of disease diagnosis and demographic characteristics, clinical manifestations and disease outcomes. Regression analysis was used to determine if SLEDAI at the onset of disease diagnosis predicts disease outcome. A two-tailed probability value (*p*-value) less than 0.05 was considered statistically significant.

## Results

Out of 902 SLE patients included in the original study, this ancillary study consisted of 823 patients as patients with incomplete data for this study were excluded. Table 1 shows the demographic, clinical, laboratory and treatment data of the study population.

**Table 1:** Demographic data, clinical manifestations and medications used for the included patients

Parameter	Value
Age at study time *mean* ± *SD* (years)	32.31 ± 9.25
Age at disease onset *mean* ± *SD* (years)	23.29 ± 8.91
Disease duration *mean* ± *SD* (years)	9.04 ± 6.10
Sex female *n* (%)	756 (91.9%)
Constitutional manifestations *n* (%)	613 (74.5%)
Mucocutaneous manifestations *n* (%)	727 (88.3%)
Musculoskeletal manifestations *n* (%)	756 (91.9%)
Hematological *n* (%)	784 (95.3%)
Thrombosis (total) *n* (%)	135 (16.4%)
Secondary vasculitis *n* (%)	214 (26.0%)
Pulmonary manifestations *n* (%)	439 (53.3%)
Cardiac manifestations *n* (%)	221 (26.9%)
Renal manifestations *n* (%)	561 (68.2%)
Neurological manifestations *n* (%)	375 (45.6%)
GIT and hepatic manifestations *n* (%)	170 (20.7%)
SLEDAI at onset of disease diagnosis (*mean* ± *SD*)	12.96 ± 8.32
SLICC (*mean* ± *SD*), range	1.57 ± 1.79, (0–10)
CCI (*mean* ± *SD*)	1.67 ± 1.54
Mortality *n* (%)	115 (14.0%)
Azathioprine *n* (%)	635 (77.2%)
Oral steroid intake *n* (%)	799 (97.1%)
Pulse methylprednisolone *n* (%)	632 (76.8%)
Cyclophosphamide *n* (%)	455 (55.3%)
Mycophenolate Mofetil *n* (%)	215 (26.1%)

*SD* standard deviation, *HTN* hypertension, *GIT* gastrointestinal manifestations, *SLEDAI* Systemic Lupus Erythematosus disease activity index, *SLICC* Systemic Lupus International Collaborating Clinics damage index, *CCI* Charlson Comorbidity Index

Regarding the studied groups, group I (those without initial severe disease) consisted of 373 patients (45.3%), and group II (those with initial severe disease) consisted of 450 patients (54.7%). The two studied groups were compared regarding the different demographic data, clinical manifestations and treatment (Table 2).

**Table 2:** Comparison of the two studied groups according to different demographic data, clinical manifestations and medications used outcome measures

	Group I (*n* = 373)	Group II (*n* = 450)	*P* value
Age at disease onset *mean* ± *SD*	24.44 ± 9.64	22.3 ± 8.31	0.001*
Sex females *n* (%)	337 (90.3)	419 (93.1)	0.15
Constitutional *n* (%)	258 (69.2)	355 (78.9)	0.001*
Mucocutaneous *n* (%)	310 (83.1)	417 (92.6)	< 0.001*
Musculoskeletal *n* (%)	328 (87.9)	428 (95.1)	< 0.001*
Cardiac *n* (%)	85 (22.8)	136 (30.2)	0.02*
Pulmonary *n* (%)	186 (49.9)	253 (56.2)	0.07*
Renal *n* (%)	222 (59.5)	339 (75.3)	< 0.001*
GIT *n* (%)	72 (19.3)	98 (21.7)	0.38
Neurological *n* (%)	117 (31.4)	258 (57.3)	< 0.001*
Haematological *n* (%)	352 (94.4)	432 (96)	0.27
Secondary vasculitis *n* (%)	76 (20.4)	139 (30.9)	0.002*
Azathioprine *n* (%)	260 (69.7)	375 (83.3)	< 0.001*
Mycophenolate Mofetil *n* (%)	90 (24.1)	125 (27.8)	0.27
Cyclophosphamide *n* (%)	170 (45.6)	285 (63.3)	< 0.001*
Pulse methylprednisolone *n* (%)	254 (68.1)	368 (81.8)	< 0.001*
SLICC	1.21 (1.61)	1.87 (1.88)	< 0.001*
CCI	0.34 (0.47)	0.42 (0.49)	0.02*
Mortality *n* (%)	48 (12.9)	67 (14.9)	0.41

*SD* standard deviation, *GIT* gastrointestinal manifestations, *n* number, % percentage, *SLEDAI* Systemic Lupus Erythematosus disease activity index, *SLICC* Systemic Lupus International Collaborating Clinics Damage Index, *CCI* Charlson Comorbidity Index

*indicates statistical significance

Correlation of SLEDAI at the disease onset with different disease parameters and outcomes was done. Patients who were younger at the onset of disease diagnosis had a statistically significant higher SLEDAI score, but it did not correlate with age at study time. SLEDAI score at the onset of disease diagnosis was correlated with damage (calculated by SLICC) and comorbidities (measured by CCI) but not with mortality (Table 3).

**Table 3:** Correlation of SLEDAI at onset of disease diagnosis with different demographic data, clinical manifestations and outcome measures

	*R*	*P* value
Age at disease onset	− 0.128	< 0.001*
Age at study time	− 0.045	0.19
Cumulative steroid dose	0.16	< 0.001*
SLICC	0.209	< 0.001*
CCI	0.195	< 0.001*
Mortality	0.027	0.44

*SLEDAI* Systemic Lupus Erythematosus Disease Activity Index, *SLICC* Systemic Lupus International Collaborating Clinics Damage Index, *CCI* Charlson Comorbidity Index

Using a regression analysis model, SLEDAI at the onset of disease diagnosis was found to be a predictor of disease damage when adjusted to age, sex, disease duration and cumulative steroid dose (Table 4).

**Table 4:** SLEDAI at onset of disease diagnosis as a predictor of damage

Outcome	Predictor variable	*B*	*Se*	*T*	95% *CI*	*P* value
Damage	Age	− 0.008	0.007	− 1.13	(− 0.02, 0,005)	0.23
	Sex	0.27	0.22	1.25	(− 0.12, 0.73)	0.16
	Disease duration	0.05	0.01	4.36	(0.02, 0.06)	< 0.001*
	Cumulative steroid dose	0.16	0.02	8.65	(0.12,0.19)	< 0.001*
	SLEDAI at the onset of disease diagnosis	0.54	0.12	4.46	(0.02,0.05)	< 0.001*

*B* unstandardized B coefficient, *Se* standard error, *t t* statistics, *CI* confidence interval, *SLEDAI* Systemic Lupus Erythematosus Disease Activity Index

*statistically significant

**Fig. 1:**
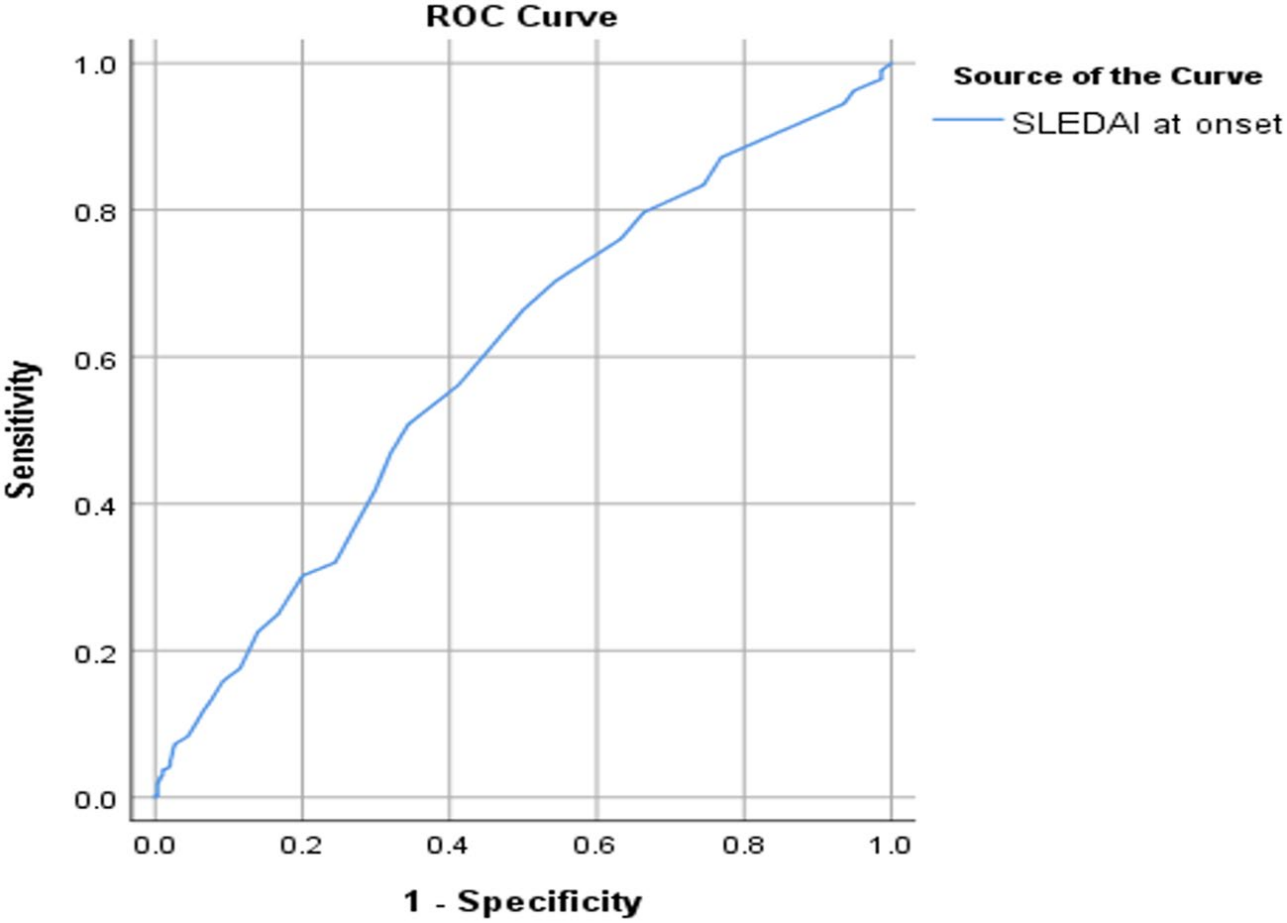
The receiving operator curve (ROC) showing SLEDAI at onset of disease diagnosis as a predictor of disease damage calculated by SLICC

**Fig. 2:**
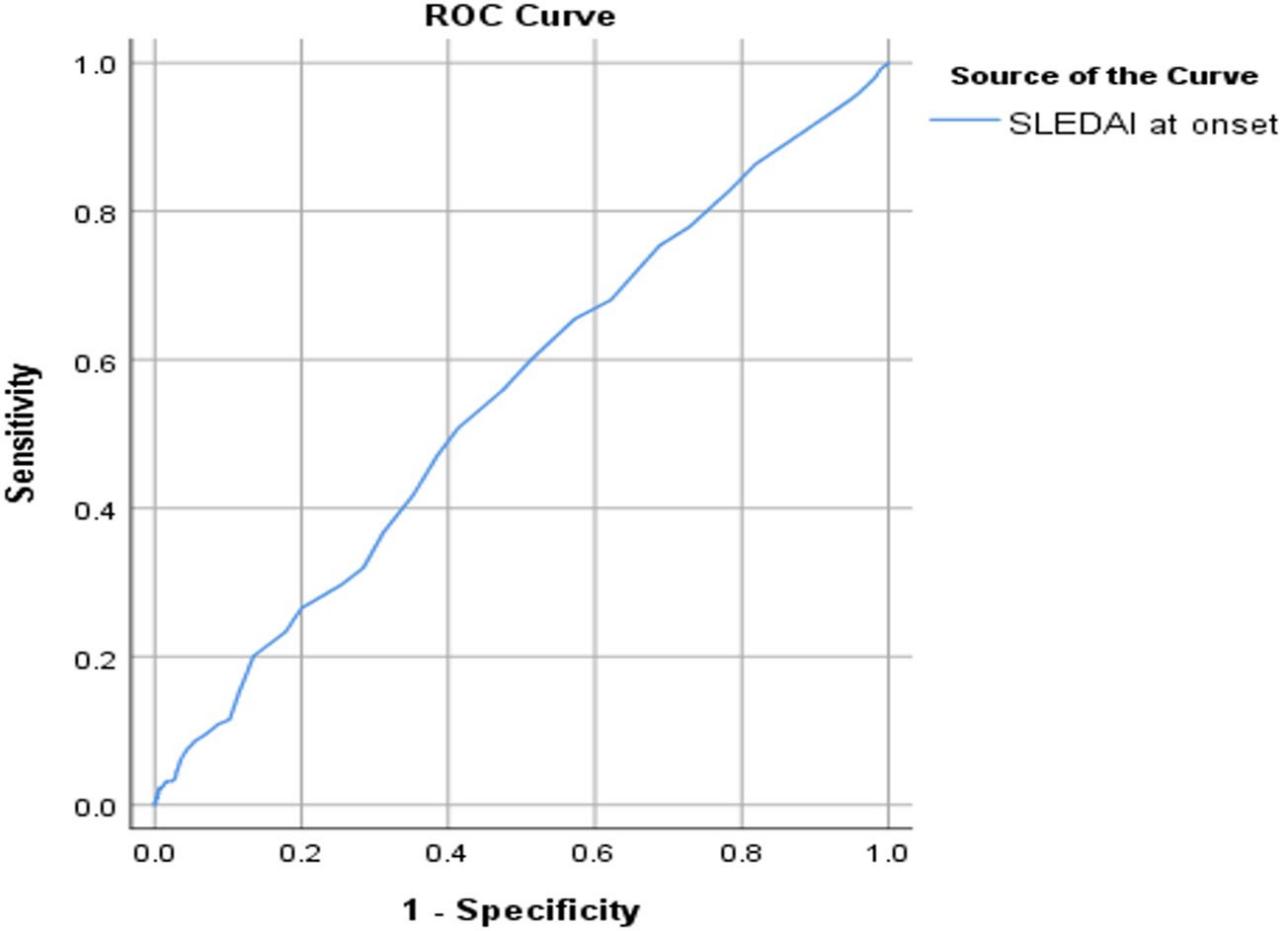
The receiving operator curve (ROC) showing SLEDAI at the onset of disease diagnosis as a predictor of comorbidities calculated by CCI

The receiving operator curve was also used to test the ability of SLEDAI at the onset of disease diagnosis as a predictor of disease damage. The area under the curve was acceptable at 0.6. A value of 13 was a cut-off value with the best sensitivity and specificity (the highest Youden index) (Fig. 1). The ability of SLEDAI at the onset of disease diagnosis as a predictor for comorbidities was tested, and the area under the curve was calculated as 0.55 with the best cut-off 13 (Fig. 2).

## Discussion

Disease activity measurement is an integral part of recommendations for disease management in patients with SLE [17, 18]. Although there are several validated disease activity indices [19, 20], SLEDAI has been widely used [19, 21] and is sensitive to changes in response to patient treatment and disease course [22, 23]. This is why we chose the SLEDAI score to measure the SLE disease activity in this study.

Our study used a disease activity score of ≥ 10 as a classification point for severe and non-severe diseases [14] which was similar to the findings of other studies [24–26].

In this study, 54.3% of patients had severe disease activity at the onset of disease diagnosis. Other studies reported similar results, as they reported that about 44% of patients in their studies had severe disease activity [24, 26, 27]. Another study found that 62% of patients had severe disease activity. These mild differences may be due to the different demographic data of the patients [28].

In this study, patients with a younger age at the onset of disease diagnosis had a higher SLEDAI score at disease onset. A similar result was reported [26].

Our study shares some similarities with other studies [24, 27], namely that patients with the following clinical manifestations have higher SLEDAI scores: constitutional, mucocutaneous, musculoskeletal, cardiac, renal, neurological and secondary vasculitis. Conversely, another study revealed a lower SLEDAI score with mucocutaneous, musculoskeletal and haematological involvement [27].

Our study coincides with some studies which stated that patients with severe disease activity have higher use of pulse methylprednisolone, cyclophosphamide and azathioprine [24, 29].

Several data points demonstrated an association between SLE disease activity and chronic damage development, regardless of the index used to assess disease activity [30]. In line with the findings of many studies, we found that patients with severe disease activity at the onset of disease diagnosis are more susceptible to organ damage than patients with non-severe disease activity [24, 29, 31–33].

In this study, severe disease activity at the onset of disease diagnosis was associated with a higher occurrence of comorbidities, which is considered an additional burden on SLE patients. This may be explained by the higher need to use more aggressive drugs that could have more side effects to control severe disease activity at the onset of disease diagnosis. For example, the use of high doses of steroids could lead to the occurrence of hypertension and diabetes mellitus.

We found a higher incidence of mortality in patients with severe disease activity at onset than in those without (67 vs. 48) but with no statistically significant difference between the two groups. A similar finding that high persistent disease activity (SLEDAI scores > 10) rather than a high initial SLEDAI is independently associated with decreased survival was reported [28]. This was reported in another study but with a statistically significant difference [34]. This difference may be because we used a cut-off value of ≥ 10 of SLEDAI to classify the disease severity while they used a different cut-off value (≥ 20).

SLEDAI at the onset of the disease diagnosis was not only correlated to disease damage but was also a fair predictor of damage in this group of patients with SLE when adjusted to other factors as age, sex, disease duration and cumulative steroid dose. SLEDAI score ≥ 13 is considered the best cut-off value to predict damage. This reflects the prognostic value of measuring SLEDAI at the onset of disease diagnosis, as it has the potential to be used outside the clinical trial setting in the identification of patients who are at higher risk of adverse outcomes. In addition, SLEDAI at the onset of disease diagnosis has a fair ability to predict comorbidities, with the best cut-off of 13.

### Strengths and limitations of this study

Our study has some strengths; to the best of our knowledge, this is the first study that focuses on the impact of disease activity at the onset of SLE disease diagnosis on different disease parameters and outcomes. Another potential strength of this post hoc analysis is the large sample size, recruited from several centres, which increases the external validity and generalizability of the results. One of the limitations of this study was that we were not able to determine the time lapse from the beginning of disease manifestations and the disease diagnosis.

### Recommendations

Confirmatory prospective studies using different disease activity measures are required to determine the most suitable disease activity score that predicts further SLE disease outcomes. Severe disease at the onset of disease diagnosis is not a modifiable risk factor, but it is important to treat it timely and aggressively with the proper therapeutics to achieve better outcomes. Special care should be directed towards patients who are younger at disease onset as they may have a higher disease activity at diagnosis and worse outcomes.

### Conclusion

A high SLEDAI score at the onset of disease diagnosis was associated with damage accrual, and this association remained after adjustment for patients’ demographic characteristics and their treatments, but it was not significantly associated with mortality. SLEDAI score 13 at the onset of disease diagnosis is considered the best cut-off value to predict damage. Severe disease activity at the onset of disease diagnosis was also associated with the occurrence of future comorbidities. Patients who are younger at disease onset are more likely to have more severe disease at the onset of disease diagnosis. Measuring SLEDAI at the onset of disease diagnosis could be a prognostic marker for predicting the damage and helping in the identification of patients who are at higher risk of adverse outcomes. Early disease diagnosis as well as early and proper therapeutic interventions should be performed. Special care should be directed towards patients who are younger at disease onset as they may have a higher disease activity at diagnosis.
